# A Useful MRI Classification for Symptomatic Discoid Lateral Meniscus

**DOI:** 10.1186/s43019-021-00108-0

**Published:** 2021-09-09

**Authors:** Eui Yub Jung, Seongmin Jeong, Sun-Kyu Kim, Sung-Sahn Lee, Dong Jin Ryu, Joon Ho Wang

**Affiliations:** 1 Department of Orthopedic Surgery, National Medical Center, Seoul, South Korea; 2 Department of Orthopaedic Surgery, Ilsan Paik Hospital, Inje University School of Medicine, Goyang-si, Gyeonggi-do South Korea; 3 Department of Orthopedic Surgery, Inha University Hospital, Inhan University School of Medicine, Incheon, South Korea; 4 Department of Orthopedic Surgery, Samsung Medical Center, Sungkyunkwan University School of Medicine, 81 Irwon-ro Gangnam-gu, Seoul, 06351 South Korea

**Keywords:** Discoid meniscus, magnetic resonance imaging, classification, meniscal tear

## Abstract

**Purpose:**

The purpose of this study is to classify the discoid lateral meniscus (DLM) according to the signal and shape in magnetic resonance imaging (MRI), and to provide information not only in diagnosis but also in treatment.

**Materials and Methods:**

We reviewed 162 cases who diagnosed with DLM by MRI and underwent arthroscopic procedures from April 2010 to March 2018. Three observers reviewed MRI findings of all cases and predicted arthroscopic tear using three MRI criteria (criterion 1,2 and 3). Among three criteria, the criterion that most accurately predicts arthroscopic tear was selected. Using this criterion, the cases of predicted tear were named group 1. In addition, group 1 was divided into three subgroups (group 1a, 1b and 1c) by deformation or displacement on MRI and arthroscopic type of tear and procedures were analyzed according to these subgroups.

**Results:**

The intra-meniscal signal change itself (criterion 3) on MRI showed the highest agreement with the arthroscopic tear. No meniscal deformation and displacement on MRI (group 1a) showed no specific type of tear and more cases of meniscal saucerization. The meniscal deformation on MRI (group 1b) showed more simple horizontal tears and more cases of meniscal saucerization. The meniscal displacement on MRI (group 1c) showed more peripheral tears and more cases of meniscal repair and subtotal meniscectomy. Comparing arthroscopic type of tear and type of arthroscopic procedure between three subgroups, there were significant differences in three groups (*P* < .05).

**Conclusions:**

Intra-meniscal signal change itself on MRI is the most accurate finding to predict arthroscopic tear in symptomatic DLM. In addition, subgroup analysis by deformation or displacement on MRI is helpful to predict the type of arthroscopic tear and procedures.

## Introduction

Discoid lateral meniscus (DLM) is a congenital variant with a prevalence of about 0.5% in whites and 15% in Asian populations [1–4]. Many patients with DLM may remain asymptomatic. Symptomatic DLM is usually associated with tears or instability, and symptomatic DLM is known to require treatment [5, 6]. In the treatment, central partial meniscectomy (saucerization) and repair as needed have shown to be more successful than total meniscectomy [7–9].

Magnetic resonance imaging (MRI) is essential for the diagnosis of DLM. MRI provides clear criteria for the diagnosis of DLM and has high specificity and sensitivity [10–12]. However, few studies have suggested MRI findings that can predict a DLM tear. The DLM is thick and occupies most of the lateral compartment and is deformed in many cases, so the arthroscopic view is poor. Therefore, the preoperative MRI findings that can accurately predict the arthroscopic tear are essential.

Ahn et al. [13] proposed a MRI classification of DLM tear by introducing the concept of meniscal shift, which provided important information for predicting the location of the peripheral tear and determining the surgical procedure. However, this classification was limited to the peripheral tear and its test property was not good (accuracy, 72.4%; sensitivity, 65.8%; specificity, 78.9%). Yoo et al. [14] reported that there is a higher probability of tear if there is a morphologic change of DLM on MRI. However, because the test property of this classification was not good (accuracy, 77%; sensitivity, 81%; specificity, 63%), they proposed that MRI should be a tool that can be complemented with physical examination and arthroscopic findings to diagnose DLM tear. This classification also did not provide useful information for surgeon to decide on the arthroscopic procedure.

In normal meniscus, signal changes localized within the meniscus on MRI are less related to tear, and it is known that signal changes are associated with tear only when they are extended into articular surface on MRI [15, 16]. However, Hameda et al. [17] reported that even with signal changes confined to the meniscus, DLM is associated with intrasubstance tear and the DLM is vulnerable to tear because the collagen arrangement is irregular and scarce than the normal meniscus [18]. Based on these results, we assumed that intra-meniscal signal change itself on MRI in DLM could predict arthroscopic tear well.

The purpose of this study is to find out most accurate MRI findings that can predict the arthroscopic tear, and to suggest an MRI classification that can predict the type of arthroscopic tear and procedures.

## Material and Method

### Patients

This study was approved by the institutional review board. The inclusion criteria of this study were as follows: the patients 1) with symptoms such as pain, swelling, limitation of motion, clicking or locking, 2) diagnosed as discoid lateral meniscus on MRI and 3) underwent the arthroscopic procedure. The exclusion criteria of this study were as follows: 1) MRI taken at other hospitals, 2) more than 6 months after MRI to arthroscopic procedure, 3) not available to arthroscopic video, 4) concomitant surgery and 5) previous surgery for discoid meniscus at the same side.

**Fig. 1:**
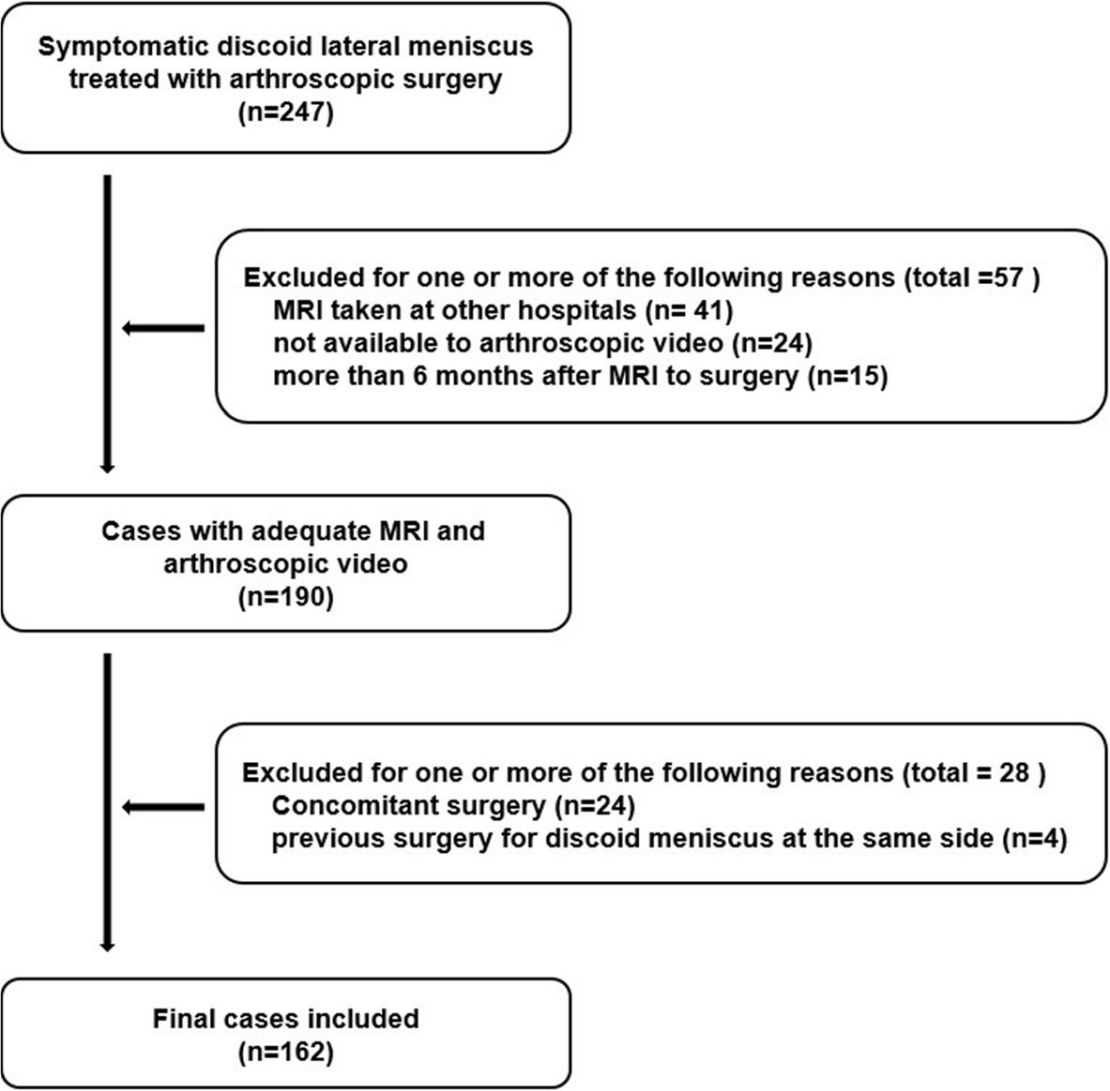
The flowchart shows patient selection and exclusion criteria

From April 2010 to March 2018, a total of 217 patients (247 knees) satisfied the inclusion criteria and 140 patients (162 knees) were included in the final analysis using the exclusion criteria (Fig. 1). The mean age at surgery was 30.1 years (range 7 to 69 years). There were 47 men (52 knees) and 93 women (110 knees). There were 64 right knees and 54 left knees and 22 bilateral knees. The 134 knees had complete DLM and 28 cases had incomplete DLM. The mean interval from the time of MRI to the time of arthroscopic surgery was 59.6 days (range, 0 to 178 days).

### Arthroscopic evaluation

Arthroscopic procedure was performed on DLM with persistent symptoms despite conservative treatment for at least 3 months after symptoms. All procedures were done under general anesthesia, except when the patient desired regional anesthesia. Arthroscopic procedures were performed using anterolateral, anteromedial and superolateral portal and if repair of posterior horn was necessary, posterolateral portal was also made. In arthroscopic examination, we checked the pattern of tear and peripheral stability using a probe, and then central partial meniscectomy (saucerization) was performed, leaving at least 6–8 mm in the peripheral portion to reshaping close to the shape of the normal meniscus [19]. In particular, when there is no tear of the meniscus surface, the portion to be excised is pre-pressed on the meniscus surface using the probe to prevent inadequate saucerization. After saucerization, the peripheral instability was checked again. If the peripheral tear was greater than 10 mm and unstable, it was repaired using all-inside, outside-in, inside-out techniques depending on its location [20–22]. Evaluation of arthroscopic video was performed with one orthopedic surgeon blinded to preoperative MRI findings. Arthroscopic types of tear were divided as follows: no tear, horizontal tear, peripheral tear, central-hole tear, radial tear and other tear. Peripheral tear refers to the separation of the meniscus at the menisco-capsular junction. Central-hole tear refers to a tear in the center of the discoid meniscus without peripheral tear. Other tears included longitudinal tear without peripheral tear, flap tear, and severe loss of meniscus.

### MRI evaluation

MRI was performed on a 3.0 T MR scanner (Achieva; Philips Medical Systems, Best, The Netherlands). We used the proton density weighted (PDW) turbo spin echo (TSE) images in the sagittal plane and 3D PDW volumetric isotropic TSE acquisition (VISTA) images in the coronal plane. The slice thickness and spacing between slices were 1.5 mm and 1.5 mm for PDW TSE sagittal images, respectively and 1.0 mm and 0.5 mm for 3D PDW VISTA coronal images, respectively. The range of repetition time (TR) and echo time (TE) varied (5000 ~ 7000 milliseconds and 20 ~ 30 milliseconds, respectively for PDW TSE sagittal images; 1600–1800 milliseconds and 30 ~ 35 milliseconds, respectively for 3D PDW VISTA coronal images). The other imaging parameters were as follows: matrix, 512 × 512; field of view, 160 mm; and number of signals averaged, 1.

**Fig. 2:**
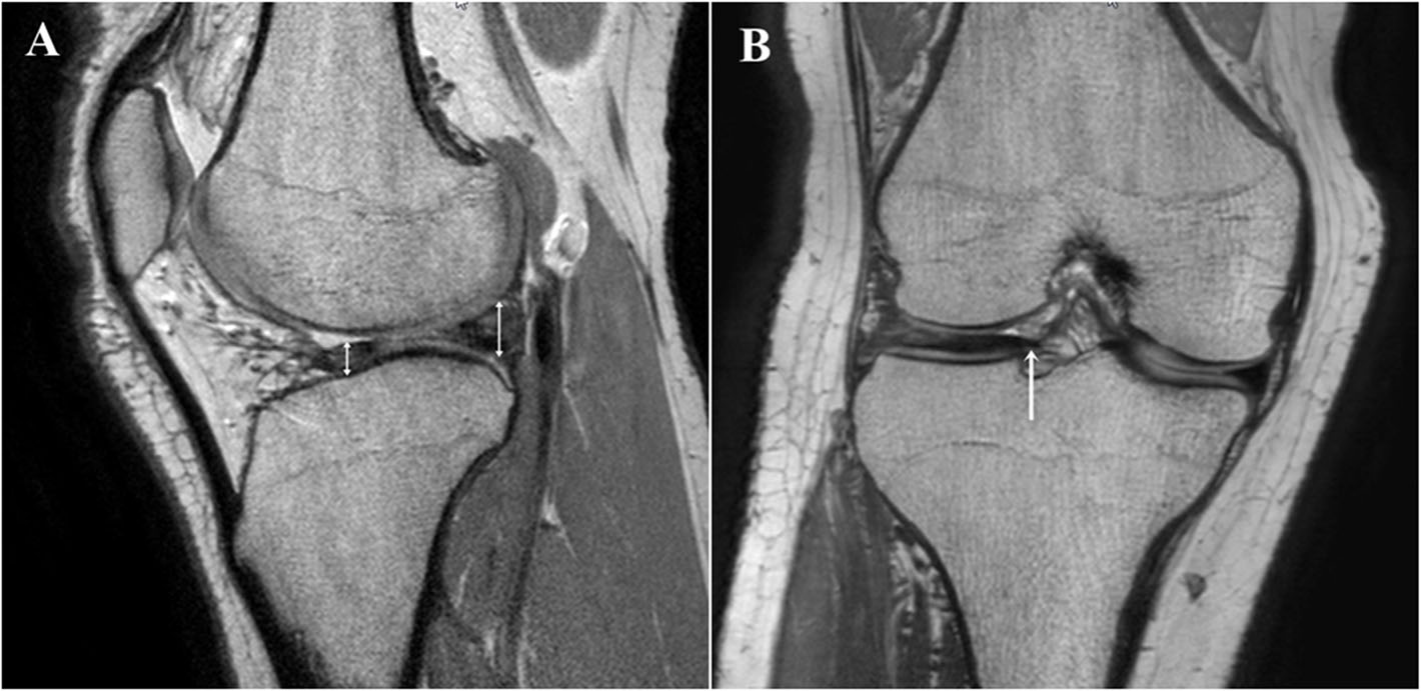
Deformation of discoid lateral meniscus on MRI. (A) the meniscus shows a greater than 70% difference of anteroposterior thickness in sagittal image (*two sided arrows*: thickness of meniscus). (B) the meniscal segment crosses the lateral tibial spine in coronal image (*arrow*)

**Fig. 3:**
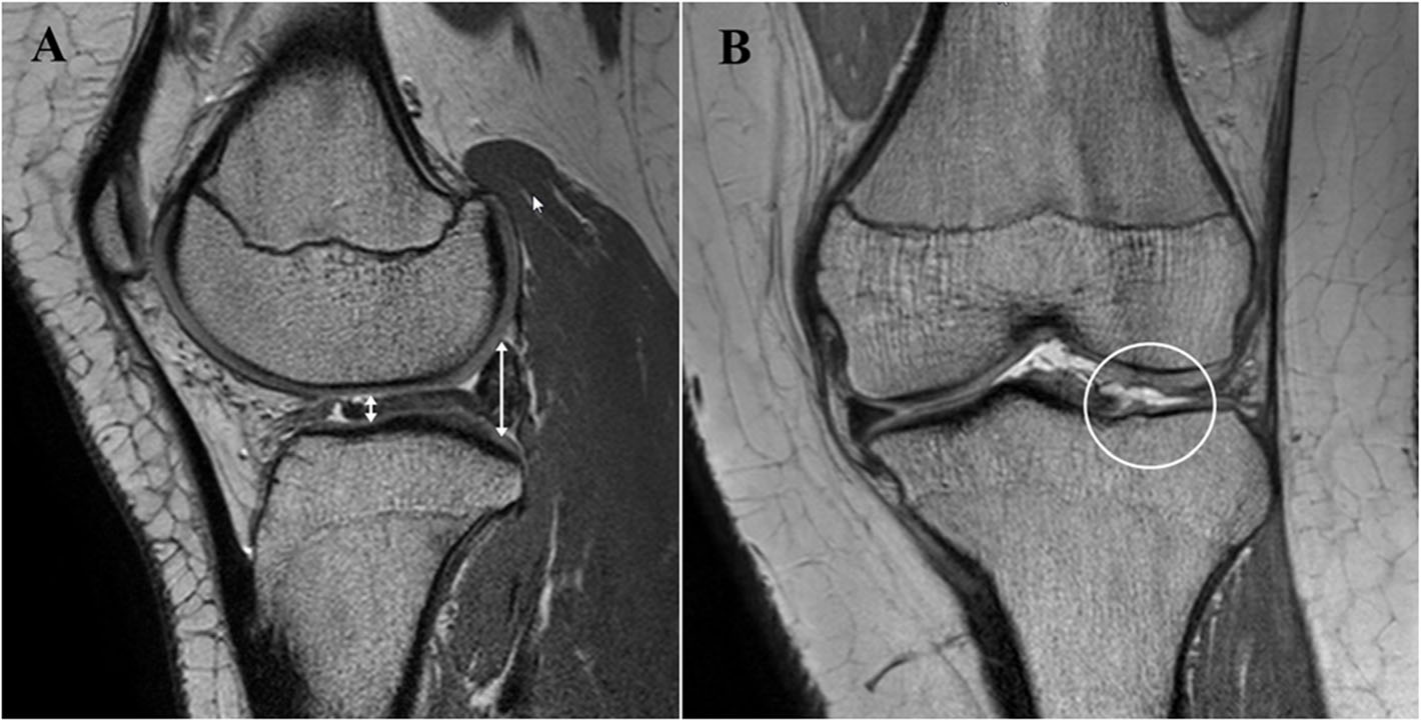
Displacement of discoid lateral meniscus on MRI. (A) the difference of thickness between the anterior and posterior horn is more than 2-fold increase in sagittal image (*two sided arrows*: thickness of meniscus). (B) the signal loss is observed on anterior side of coronal image (*empty circle*: detached side of meniscus)

Three experienced orthopedic surgeons checked MRI findings of all case, blinded to arthroscopic findings. When checking MRI findings, only the patient’s registration number was informed, and images without patient’s information were checked on the Picture Archiving and Communication System (PACS). Three orthopedic surgeons used 3D PDW VISTA coronal image and PDW TSE sagittal image to predict the presence of DLM tear. They predicted DLM tear according to the following three MRI criteria. Criterion 1 defined the tear as the cases with linear or band like signal intensity extending to the superior or inferior articular surface, which was used in normal meniscus [15, 23]. Criterion 2 defined the tear as the cases of deformation or displacement of the meniscus. Deformation was defined 1) when the peripheral portion of the discoid meniscus did not detach from the capsule and the entire meniscus was not displaced and 2) when meniscus showed a greater than 70% difference of anteroposterior thickness in sagittal image and mediolateral thickness in coronal image or 3) When meniscus segment crossed the lateral tibial spine in coronal image or crossed the margin on tibia plateau in sagittal image [14] (Fig. 2). Displacement was defined 1) when the signal loss was observed in more than 4 cuts (6 mm) on anterior or posterior side of coronal images or 2 cuts (3 mm) on peripheral side of coronal images and 2) when the difference of thickness between the anterior and posterior horn was more than 2-fold increase in sagittal image [13] (Fig. 3). Criterion 3 defined the tear as a signal change only within the meniscus irrespective of the deformation and displacement of the meniscus.

With the results of checking by three orthopedic surgeons for each criterion, the inter-observer reliability of three orthopedic surgeons was measured. Intra-observer reliability for each orthopedic surgeon was measured using the same MR images after 2 weeks. If three orthopedic surgeons made different predictions for the DLM tear, the results of two orthopedic surgeons who made the same prediction were followed.

### Subgroup analysis

We selected the most accurate and reliable MRI criterion for predicting arthroscopic tear using statistical tests. Using this criterion, the cases of predicted tear were named group 1 and divided into three subgroups as follows: 1) group 1a refers to cases of no deformation and displacement, 2) group 1b refers to the cases of deformation and 3) group 1c refers to the cases of displacement. Three orthopedic surgeons have done subgrouping on MRI respectively. The distribution of arthroscopic type of tear and type of arthroscopic procedure were compared between these subgroups.

### Statistical analysis

**Fig. 4:**
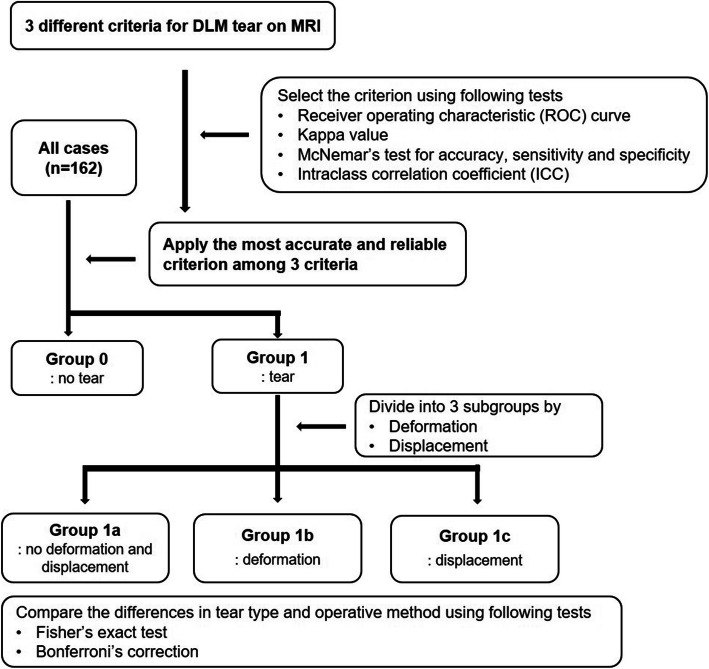
The flowchart shows how all cases were classified. (*DLM* discoid lateral meniscus)

In this study, the following statistics was used to derive MRI classification for DLM tear (Fig. 4). The intra-class correlation coefficient (ICC) was used to identify inter-observer and intra-observer reliability for determining the presence of DLM tears and for dividing into three subgroups. Receiver operating characteristic (ROC) curves were analyzed to determine how much of the three DLM tear criteria on MRI could predict the arthroscopic tear, and the DeLong’s test was used to confirm whether there was a significant difference between the ROC curves. The agreement between the presence of tear on MRI and arthroscopic tear on the three criteria was checked by Kappa (*κ*) value. The accuracy, sensitivity, specificity, positive predictive value (PPV) and negative predictive value (NPV) were also analyzed and McNemar’s test or Bennett’s test was used for multiple comparisons analysis of the three criteria for these statistical results. The differences between the arthroscopic type of tear and type of arthroscopic procedure between subgroups were analyzed by Fisher’s exact test and the *p* value correction of multiple comparisons analysis was performed with Bonferroni’s correction. All tests were two-sided tests and statistical tests were considered significance at *P <* .05. Statistical analysis was executed using SAS version 9.4 (SAS Institute, Cary, NC) and R 3.4.4 (Vienna, Austria; http://www.R-project.org/).

## Results

The ICCs of intra- and inter-observer reliability for criterion 1 were 0.745 and 0.673, respectively, those for criterion 2 were 0.669 and 0.784 and those for criterion 3 were 0.817 and 0.906. There was no significant difference between the three ROC curves (criterion 1 and 2, *P* = .288; criterion 1 and 3, *P* = .093; criterion 2 and 3, *P* = .322) and the area under curves (AUC) of criterion 1, 2 and 3 were 0.795, 0.742, and 0.693, respectively.

According to criteria of Landis and Koch [24], the *κ* values of criterion 1 and 2 were 0.337 and 0.367, respectively with fair agreement and the *κ* value of criterion 3 was 0.447 with moderate agreement. The accuracy, sensitivity, specificity, PPV and NPV for criterion 1 were 75.93%, 74.83%, 84.21%, 97.27% and 30.77%, respectively. Those for criterion 2 were 82.72%, 85.31%, 63.16%, 94.57%, 36.36% and those for criterion 3 were 90.12%, 96.50%, 42.11%, 92.62%, 61.54%. Multiple comparisons test of the three criteria for accuracy, sensitivity, and specificity showed significant differences in all comparisons (*P* < .05). But those for PPV and NPV showed significant differences only in some comparisons (criterion 1 and 2, *P* = .081; criterion 1 and 3, *P* = .017; criterion 2 and 3, *P* = .137 for PPV, criterion 1 and 2, *P* = .250; criterion 1 and 3, *P* = .039; criterion 2 and 3, *P* = .058 for NPV) (Table 1). Of the three criteria, the criterion 3 showed the highest ICC, *κ* value, accuracy, and sensitivity and we choose the criterion 3 as the most accurate and reliable criterion for predicting arthroscopic tear.

**Table 1:** Test property to predict actual tear by MRI criteria

	Criterion 1	Criterion 2	Criterion 3	*p* value
Kappa value	0.337	0.367	0.447	
Test property				
Accuracy	75.93	82.72	90.12	< 0.05 ^a^
Sensitivity	74.83	85.31	96.50	< 0.05 ^a^
Specificity	84.21	63.16	42.11	< 0.05 ^a^
PPV	97.27	94.57	92.62	> 0.05 ^b^
NPV	30.77	36.36	61.54	> 0.05 ^b^
Intraclass correlation coefficient				
Intra-observer	0.745	0.669	0.817	< 0.05
Inter-observer	0.673	0.784	0.906	< 0.05

Significance was calculated using a two-tailed, ^a^ McNemar’s test and ^b^ Bennett’s test

Values of test property are presented as the percentage (%)

*PPV* positive predictive value, *NPV* negative predictive value

The ICCs of intra- and inter-observer reliability for subgrouping were 0.915 and 0.854, respectively. There was a significant difference between the three subgroups in the distribution of arthroscopic types of tear (*P* < .05). In multiple comparisons analysis, the distribution of simple horizontal tear was significantly higher in group 1b than in the other two subgroups (14.3%, 41.7%, 1.7% for group 1a, 1b and 1c, respectively) and that of peripheral tear was significantly higher in group 1c than in the other two subgroups (18.4%, 11.1%, 71.9% for group 1a, 1b and 1c, respectively). That of central hole tear, radial tear and other tear were no significant differences between the three subgroups. There was a significant difference between the three subgroups in the distribution of type of arthroscopic procedure (*P* < .05). In multiple comparisons analysis, the distribution of cases of meniscal saucerization was significantly higher in group 1a and 1b than group 1c (75.5%, 80.5%, 17.2% for group 1a, 1b and 1c, respectively). That of meniscal repair and subtotal meniscectomy were significantly higher in group 1c than in the other two subgroups (24.5%, 16.7%, 62.5% for meniscal repair and 0.0%, 2.8%, 20.3% for subtotal meniscectomy for group 1a, 1b and 1c, respectively) (Table 2).

**Table 2:** Distribution of arthroscopic tears and procedures by subgroups

	Group 1a (*n* = 49)	Group 1b (*n* = 36)	Group 1c (*n* = 64)	*p* value
Arthroscopic type of tear				
no tear	5 (10.2)	4 (11.1)	2 (3.1)	
Simple horizontal tear	7 (14.3)	15 (41.7)	1 (1.6)	< 0.05 ^a^
Peripheral tear	9 (18.4)	4 (11.1)	46 (71.9)	< 0.05 ^a^
Central-hole tear	5 (10.2)	3 (8.3)	6 (9.4)	1.00 ^a^
Radial tear	9 (18.4)	2 (5.6)	0 (0.0)	< 0.05 ^a^
Other tear	14 (28.5)	8 (22.2)	9 (14.1)	0.152 ^a^
Type of Arthroscopic Procedure				
Saucerization Only	37 (75.5)	29 (80.5)	11 (17.2)	< 0.05 ^a^
Suacerization + Repair	12 (24.5)	6 (16.7)	40 (62.5)	< 0.05 ^a^
Subtotal meniscectomy	0 (0.0)	1 (2.8)	13 (20.3)	< 0.05 ^a^

Values are presented as the number of cases (%)

Significance was calculated using a two-tailed, ^a^ Fisher’s exact and the *p* value correction of was performed with Bonferroni’s correction

## Discussion

The principal findings of the present study are that the intra-meniscal signal change itself on MRI can predict the arthroscopic tear accurately in DLM and subgroup analysis by deformation or displacement on MRI is helpful to predict the type of arthroscopic tear and procedures. The subgroups showed the distinct differences of distribution as follows: 1) When DLM show no deformation and displacement on MRI (group 1a), arthroscopic findings showed no specific types of tear and surgeons tended to perform more meniscal saucerization. 2) When DLM show only deformation, arthroscopic findings showed more simple horizontal tears and surgeons tended to perform more meniscal saucerization. 3) When DLM show only displacement (group 1c), arthroscopic findings showed more peripheral tears and surgeons tended to perform more meniscal repair or subtotal meniscectomy (Table 3).

**Table 3:** The table shows which type of tear can be predicted and which arthroscopic procedures can be predicted according to the presence or absence of deformation or displacement on MRI in discoid lateral meniscus

	Arthroscopic Type of Tear	Arthroscopic Procedure
No Deformation and Displacement (1a)	• No Specific Type of Tear	• Saucerization
Deformation (1b)	• Simple Horizontal Tear	• Saucerization
Displacement (1c)	• Peripheral Tear	• Saucerization + Repair • Subtotal Meniscectomy

Because the arthroscopic view of cases for DLM is poor, it is useful to know the presence of tear and the type of tear on preoperative MRI before surgery. However, the previous studies of DLM using the MRI were very heterogeneous in terms of criteria for tear. For example, they did not mentioned the criteria for determining the tear [25], used the criteria for tear in normal meniscus [26, 27] and most studies have applied arthroscopic types of tear in normal meniscus to the types of tear on MRI in DLM, but even it slightly different [25, 26, 28–30]. These studies also report that the type of tear on MRI in DLM is difficult to predict in many cases. In this context, this study was intended to develop an MRI classification applicable only to DLM.

Few studies have suggested the MRI classification applicable to DLM. Ahn et al. [13] presented a new classification using the concept of meniscal shift, which can give excellent information in selecting the type of arthroscopic procedure. However, this was limited to information on peripheral tear and was not accurate in predicting DLM tear (sensitivity 65.8%, specificity 78.9%, accuracy 72.4%). Yoo et al. [14] presented the classification using the concept of meniscal morphologic change, which was developed by complementing the concept of meniscal shift proposed by Ahn et al. [13] However, this classification was not reliable in predicting DLM tear (sensitivity of 76%, NPV of 44%) and no information about surgical procedure or type of tear was available. The classification in this study determined the presence of DLM tears based on intra-meniscal signal change itself on MRI and could show reliable test properties (accuracy, sensitivity, specificity, PPV and NPV; 90.12%, 96.50%, 42.11%, 92.62% and 61.54%). In addition, the type of tear and type of arthroscopic procedure showed distinct difference when divided into three subgroups by modifying the concept presented in the previous study.

In normal meniscus, it is known that when signal change is localized within the meniscus, it is not related to tear [15, 16]. However, in case of DLM, Hameda et al. [17] reported that intra-meniscal high signal intensity is the evidence of intrasubstance tear or degeneration even without intra-articular extension on MRI. This is consistent with the results of this study, in which the intra-meniscal signal change itself was most related to tear on MRI in DLM. These results suggest that DLM is more susceptible to intrasubstance tear than normal meniscus. In fact, in some studies, intrasubstance tear is the most prevalent tear type in DLM [8, 31]. This may be due to the fact that the fiber arrangement inside the DLM is less organized and scarce than the normal meniscus [18, 32, 33]. The fact that horizontal cleavage tear is more common in complete DLM than incomplete DLM [28, 31] can be considered as indirect evidence that DLM is vulnerable to intrasubstance tear compared to normal meniscus. In this study, simple horizontal tear also was more common in complete DLM (17.9%, 24/134 cases) than incomplete DLM (7.1%, 2/28 cases).

In this study, using the concept of deformation and displacement, dividing the group of tear into subgroups could give information about the distribution of arthroscopic type of tear and type of arthroscopic procedure. The deformation is a modification of the concept of morphologic change introduced by Yoo et al. [14], except that it is shifted by peripheral detachment and the displacement is a modification of the concept of meniscal shift introduced by Ahn et al. [13], including the change of criteria of signal loss from 10 mm to 6 mm and the concept of signal loss on the lateral side. In this study, we used these concepts for subgrouping because it was thought that these concepts would be more useful for informing about the type of tear and type of arthroscopic procedure than determining the presence or absence of tear. In fact, Yoo et al. [14] reported that meniscal morphologic change on MRI (nearly the same as deformation) occurred in 77% of isolated horizontal cleavage tears on arthroscopy, and Ahn et al. [13] presented that meniscal shift on MRI (nearly same as displacement) was closely related to peripheral tear and meniscal repair or subtotal meniscectomy. Similar to the results of previous studies, in this study, horizontal tears were common in group of deformation (group 1b), and peripheral tear, meniscal repair and subtotal meniscectomy were more frequent in group of displacement (group 1c). In addition, the group of no deformation and displacement (group 1a) showed no specific type of tear, but meniscal reshaping was common. These results are meaningful because it is possible to know approximately the information about the arthroscopic type of tear and type of arthroscopic procedure by only the change of DLM’s shape on MRI.

However, this classification has some limitations. First, the specificity was low (41.2%) when the DLM tear was determined by intra-meniscal signal change itself on MRI. In other words, the false positive rate is high. It is possible that meniscal degeneration may have caused symptoms before intrasubstance tear and in the case of skeletal immature patients, the false positive of MRI may be high due to the high vascularity of meniscus. Generally, all of the arthroscopic procedures performed in the DLM are symptomatic DLM cases and arthroscopic procedures can be fully understood if symptoms are present, even if false positive cases because symptomatic DLM is commonly treated with surgical interventions [5, 6]. In this study, this situation also occurred in group 0 (arthroscopically no tear). It may be meaningful to examine the degeneration or vascularity of the meniscus by histologic evaluation of excised discoid meniscus in cases with symptoms but no arthroscopic tear. Second, all of the MRIs in this study were static images taken in the knee extension state. Therefore, despite the instability of the peripheral tear, the MRIs were reduced at the moment of taking the MRI, and classified as group 1a and 1b instead of group 1c. Ahn et al. [13] reported 13 cases of similar situation and advised to consider peripheral tear if physical examination findings such as loud click or clunk appear. In this study, there were peripheral tears of 13 cases out of 85 cases, group 1a and 1b. In all cases, meniscal repair was possible after the detection of peripheral tear by thorough probing after meniscal saucerization. Third, because the age distribution of the study subjects was different from those of the previous studies, the results should be interpreted with caution. Although they did not mention the reason, most previous studies on DLM classification have studied patients under 20 years of age [8, 13, 14]. But, we concluded that it is better not to limit the age of the study subjects because the distribution of patients with DLM who actually visited the hospital and underwent surgery due to symptom was varied. The mean age of the subjects in this study was 30.1 years (ranged from 7 to 69) and 64 of 162 (39.5%) were below 20 years of age and Kim et al. [28] reported an average age of 27.9 years (range 3–59 years) in the analysis of arthroscopic findings of symptomatic DLM in 164 cases and reported that the 20–30 age group accounted for 55% of the total subjects. As such, if the age limit of 20 years or less is used as in the previous studies, the representativeness of the classification may be lowered because approximately half of the symptomatic patients with DLM are excluded.

## Conclusion

In the symptomatic DLM, when the intra-meniscal signal change itself on the MRI was used as a reference for arthroscopic tear, a more accurate prediction of tear was possible than the other references. In addition, subgrouping of tears on MRI by meniscal deformation and displacement revealed that the arthroscopic type of tear and type of arthroscopic procedure showed distinct differences between subgroups. Therefore, the classification of this study can give useful information to surgeons in preparing the treatment of the symptomatic DLM.

## Data Availability

The datasets used and/or analyzed during the current study are available from the corresponding author on reasonable request.
